# Sodium Bicarbonate Can Increase Milk Fat Concentration in Lactating Ewes Fed Ryegrass Herbage Rich in Water-Soluble Carbohydrates

**DOI:** 10.3390/ani16162481

**Published:** 2026-08-10

**Authors:** Maria Angela Porcu, Antonello Ledda, Silvia Carta, Ana Helena Dias Francesconi, Antonello Cannas

**Affiliations:** Department of Agricultural Sciences, University of Sassari, 07100 Sassari, Italy; anledda@uniss.it (A.L.); scarta2@uniss.it (S.C.); france@uniss.it (A.H.D.F.); cannas@uniss.it (A.C.)

**Keywords:** buffer, milk fat reduction, fresh grass, ryegrass, water-soluble carbohydrates, milk fat, dairy sheep, dairy ruminants, sodium bicarbonate, grazing

## Abstract

Pastures rich in water-soluble carbohydrates (WSCs) can decrease milk fat concentration in dairy ruminants, associated with rumen pH reduction. To prevent milk fat reduction, sodium bicarbonate was supplemented as a rumen buffer to sheep fed indoors on tetraploid annual ryegrass (*Lolium multiflorum* Lam. ssp. westervoldicum) herbage rich in WSCs (28% DM). Ten lactating Sarda ewes were assigned to the control group (CNT) or the sodium bicarbonate group (BIC; 25 g/day per ewe before ryegrass feeding). All ewes were housed indoors, machine-milked at 16:30 and 07:30, fed freshly cut ryegrass ad libitum (12:30 to 07:30 of subsequent day), and fed concentrates during milkings. Individual grass, measured with automatic feeders, and concentrate intake were recorded. Total dry matter intake (DMI) (*p* = 0.03), ryegrass DMI (*p* = 0.03), milk fat concentration in afternoon (*p* < 0.01) and morning (*p* = 0.03) milkings, and afternoon milk fat yield (*p* = 0.03) were higher in BIC than CNT. Milk yield was unaffected. In conclusion, sodium bicarbonate supplementation can increase milk fat concentration in ewes fed high-WSC herbage, with rapid effect on the rumen. WSC concentration should be considered when formulating rations for pasture-fed dairy sheep and, more generally, dairy ruminants.

## Simple Summary

Pastures of grasses can reach high concentrations of water-soluble carbohydrates, a sum of simple sugars (mainly glucose, fructose, and sucrose) and total fructans, especially during sunny and cold days. Their concentration changes during the day and over the season, and it is influenced by plant species and cultivar as well as environmental conditions. Water-soluble carbohydrates can be associated with digestive alterations, such as decreased ruminal pH and milk fat reduction in ruminants. This study evaluated if sodium bicarbonate supplementation, administered orally as a rumen buffer, could prevent the drop in milk fat concentration in housed lactating Sarda ewes fed fresh annual ryegrass rich in water-soluble carbohydrate concentration. The results showed that sodium bicarbonate improved total intake, herbage intake, milk fat concentration, and milk fat yield. In conclusion, sodium bicarbonate supplied just before herbage intake increased milk fat concentration in dairy ewes consuming herbage high in water-soluble carbohydrates indoors.

## 1. Introduction

Modern ryegrass varieties and other C3 grasses widely used for grazing and forage production can reach extremely high water-soluble carbohydrate (WSC) concentrations (>25–30% of DM) [1,2,3]. Although high WSC levels in grasses were considered typical of high-latitude regions [4], such high values have been recently recorded also under Mediterranean conditions [1,2,5]. This is likely due to both the increased cultivation of modern grass varieties and the ongoing climate change, including an increase in the diurnal temperature range [6] and more frequent dry spells, which result in clear skies and reduced precipitation [7]. The consumption of herbage rich in WSC (i.e., simple sugars and fructans) can reduce rumen pH [2,8,9,10] and can cause dietary imbalances [10] in ruminants. Moreover, the use of high-WSC grasses has also been associated with a reduction in milk fat concentration in sheep [3,11]. Given the potential negative effects associated with high WSC concentrations, mitigation strategies for ruminants fed these types of herbage need to be developed.

Sodium bicarbonate (NaHCO_3_) is widely used, either individually or in combination with magnesium oxide, as a buffer in dairy ruminant rations [12,13] to prevent ruminal pH decline and, consequently, ruminal acidosis [12,13,14,15] in confined ruminants. However, this rumen buffer is not commonly used in grazing systems [16]. Sodium bicarbonate has a rapid buffering action on ruminal fluid, thus preventing post-prandial increases in H^+^, and it may also enhance nutrient dilution and ruminal fluid outflow [16,17]. In addition, its use in ruminants can increase dry matter intake (DMI) [14,18,19], nutrient digestibility [19,20], ruminal total volatile fatty acids (VFAs) [21,22], milk yield and milk fat content [23]. However, responses to sodium bicarbonate vary depending on diet, e.g., with greater effects observed at a lower initial ruminal pH [13,14,19].

In castrated male sheep fed fresh herbage (mixed legume and grass sward at vegetative stage) indoors, supplementation with sodium bicarbonate and magnesium oxide (75% and 25%) at 20 g/d increased ruminal fluid outflow rate and rumen pH [16]. However, in grazing dairy cows fed concentrates and high-quality pasture (grasses and clover mix), sodium bicarbonate supplementation (1.9% of the concentrate, i.e., on average 185 g/d) did not alleviate moderate milk fat depression (MFD) [24]. Unfortunately, WSC concentrations were not measured in these studies.

To the best of our knowledge, no other studies have investigated the use of buffers in high-WSC herbage-fed ruminants, especially focusing on whether sodium bicarbonate supplementation can mitigate the negative effects of high-WSC intake from herbage on milk fat content. We hypothesized that sodium bicarbonate, supplied prior to the intake of WSC-rich herbage, could limit the reduction in milk fat concentration caused by this type of herbage in ruminants. Thus, the aim of this study was to evaluate the effect of sodium bicarbonate supplementation, supplied just before the start of herbage feeding, on milk fat concentration in housed lactating Sarda ewes fed fresh tetraploid annual ryegrass with a high WSC concentration.

## 2. Materials and Methods

The animal experiment was conducted at the experimental farm of the Department of Agricultural Sciences of the University of Sassari located in the north-west of Sardinia (Ottava, Sassari, Italy; 40°48′41.4″ N 8°17′50.7″ E), from 6 to 17 April 2023 (12 animal experimental days), with two pre-experimental measurements performed on 2 and 5 April 2023. The animal protocol used was in accordance with European Union and Italian regulations on animal welfare, and all measurements were taken by personnel trained and approved by the Body Responsible for Animal Welfare and Experimentation of the University of Sassari (O.P.B.S.A).

### 2.1. Experimental Site, Animals and Diet

On the experimental farm, a field of tetraploid annual ryegrass (*Lolium multiflorum* Lam. ssp. westervoldicum) was sown at a seeding rate of 30 kg/ha on 18 October 2022. At sowing, the field was fertilized with nitrogen at 130 kg/ha, applied in the form of urea (46% N) by using a fertilizer also containing dicyandiamide (inhibitor of nitrification). During the animal experiment, the tetraploid annual ryegrass was harvested daily at 11:30 h. The plants were at the late stem elongation to early booting stage. Herbage was cut at a height of 8–12 cm from the ground, by using an Attila AT 900 MF (Brumar srl, Asti, Italy) mowing machine, and immediately taken to the barn to feed the ewes indoors. The stability over time of the WSC concentration in the herbage after harvest was previously evaluated and confirmed prior to the start of trial.

From sowing (18 October 2022) until the end of the trial (17 April 2023), weather data (minimum and maximum temperature, solar radiation, and rainfall) were recorded at the meteorological station of the experimental farm, located approximately 150 m from the ryegrass field. Mean monthly weather conditions are presented in Table 1.

The experiment had a preliminary period of adaptation to the herbage diet fed indoors of 12 days, which ended on 5 April 2023, and an experimental period of 12 days (6–17 April 2023). Ten dairy Sarda ewes in the third month of lactation were selected from a larger indoor-housed group. Based on pre-experimental average measurements of milk yield (2123 ± 254 g/ewe per day, mean ± SD; 2113 ± 367 and 2133 ± 97 g/ewe per day for CNT and BIC, respectively), body weight (58.6 ± 6.7 kg) and milk fat concentration (5.50 ± 0.46%; 5.6 ± 0.46% and 5.4 ± 0.5% for CNT and BIC, respectively), the ewes were allocated to two homogeneous groups of 5 animals per treatment each, in a complete randomized design: one control group (CNT, no sodium bicarbonate) and one treated group supplemented with sodium bicarbonate (BIC). A total of five ewes were assigned to each treatment group because the study required accurate measurements of individual fresh herbage intake and hourly feeding patterns using automatic BioControl Controlling and Recording Feed Intake (CRFI) feeders (BioControl AS, Rakkestad, Norway). The limited number of available CRFI feeders (*n* = 10), together with the need to harvest, transport, and provide an adequate quantity of homogeneous fresh herbage daily to all ewes (supplied ad libitum), constrained the experimental group size. The experimental period lasted 12 days, as sodium bicarbonate exerts a rapid buffering effect in the rumen and the animals had already been adapted to the experimental diet before treatment allocation. Indeed, ewes were adapted to the fresh herbage diet for 12 days before the start of the experimental period to ensure stable ruminal adaptation.

**Figure 1:**
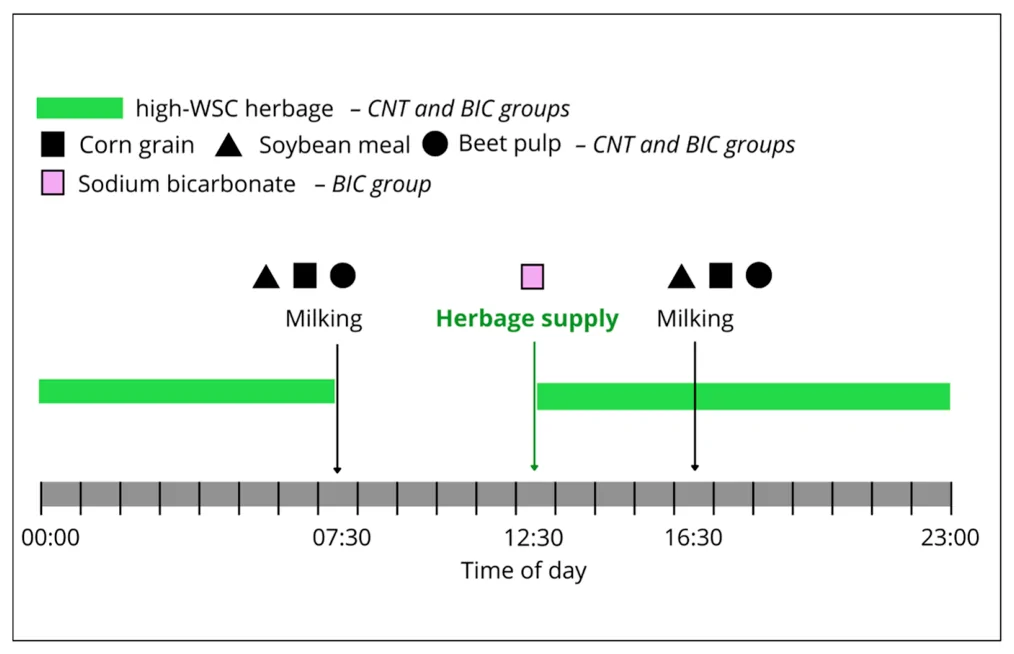
Daily feeding and milking (black arrows) regimen for the control (CNT, no sodium bicarbonate) and treated (BIC, sodium bicarbonate) ewe groups during the animal experiment (from 6 to 17 April 2023). The ewes of both groups received high-WSC annual ryegrass herbage (*Lolium multiflorum* Lam. ssp. westervoldicum) ad libitum, 550 g/day of corn grain, and 400 g/day of soybean meal throughout the trial, as well as 300 g/day of beet pulp until 7 April 2023. Herbage was cut only once a day and supplied ad libitum indoors at 12:30 (green arrow) by using automatic feeders (Biocontrol AS, Rakkestad, Norway).

All animals were housed indoors and machine-milked twice daily at 07:30 and 16:30. The ewes were housed together in a single pen bedded with wood shavings and were individually fed the ryegrass herbage ad libitum from 12:30 to 07:30 of the subsequent day, by using the ten automatic feeders (BioControl AS, Rakkestad, Norway). Animals could eat the herbage in any of the feeders, which continuously record individual intake with electronically monitored weigh cells applied under each feeder. In addition, the ewes received individually a total of 400 g/day per head of soybean meal, 550 g/day per head of whole corn grains, and 300 g/day per head of beet pulps (as-fed values), split into two separate equal meals at each milking. Intake of concentrates was recorded by manually weighing the offer and the refusals after each meal. The BIC group was also supplemented with 25 g/day per ewe of sodium bicarbonate (about 1% of total predicted DMI) just before the ryegrass supply. Sodium bicarbonate was dissolved in water and administered orally via syringe to ensure that all animals received the same amount of the supplement, whereas the CNT group did not receive any sodium bicarbonate supplementation before herbage supply. Water was provided ad libitum. The diets were planned to sustain the initial recorded average milk production and to reflect the diets used in modern high-yield, pasture-based dairy sheep systems, which combine herbage with concentrate supplementation. The ingredients and time of supply of the experimental diets, as well as the milking time, are reported in Figure 1.

### 2.2. Measurements, Sampling and Analyses

Samples of the herbage supplied to the ewes were collected daily, immediately after mowing, and were divided into two subsamples, one immediately frozen at −20 °C and the other dried immediately at 65 °C and then ground to pass through a 1 mm screen. Dry matter (DM) content was determined by oven-drying at 105 °C for 24 h. Neutral detergent fiber (NDF), acid detergent fiber (ADF), acid detergent lignin (ADL), crude protein (CP), soluble crude protein, ash, ether extract (EE) and acid detergent insoluble crude protein (ADICP) were determined by near-infrared reflectance spectroscopy (NIRS) in Cargill Laboratories (Fiorenzuola D’Arda, Italy). NFC was calculated as follows: NFC = 100 − (NDF + CP − ADICP + ash + EE). The WSC content of herbage samples was determined by the anthrone sulphate colorimetric method [25] using glucose as a standard. The absorbance value was colorimetrically determined at 625 nm.

The herbage individual intake and intake rate were monitored and recorded continuously using the automatic feeders (Biocontrol AS, Rakkestad, Norway). The following feed intake parameters were calculated: total DMI, daily grass intake, daily concentrate intake, grass intake before the afternoon milking (from 12:30 to 16:30), grass intake before the subsequent morning milking (from 17:00 to 07:30 of the subsequent day), daily WSC intake, WSC intake before the afternoon milking (from 12:30 to 16:30), WSC intake before the subsequent morning milking (from 17:00 to 07:30 of the subsequent day), NDF intake, and CP intake.

Individual milk yields (g/ewe) were measured daily at each milking (afternoon milking at 16:30 and subsequent morning milking at 07:30), using appropriate vessels to collect and weigh the milk of each ewe. Individual milk samples were collected twice a week and divided into two subsamples. One subsample was refrigerated at 4 °C and analyzed on the same day for the determination of fat and protein using a Milkoscan 6000 instrument (Foss Electric, Hillerød, Denmark); the second aliquot was frozen at −20 °C for subsequent fatty acid (FA) determination using gas chromatography (GC) analysis.

The determination of FAs started with the extraction of milk fat, which was performed according to Nudda et al. [26]. Afterwards, the preparation of fatty acid methyl esters (FAMEs), including their separation and quantification, was performed according to Correddu et al. [27], by using a 7890A GC System (Agilent Technologies, Santa Clara, CA, USA). As reported by Correddu et al. [27], the initial temperature of the oven was set to 45 °C, maintained for 4 min, and then it increased until reaching 175 °C at a rate of 13 °C/min. This temperature was held for 27 min, after which the temperature increased at 4 °C/min until reaching 215 °C, and maintained for 35 min. The identification of individual FA was performed using OpenLAB CDS GC ChemStation Upgrade software (Revision C.01.04, Agilent Technologies, Santa Clara, CA, USA) by comparing their retention times with those of methyl ester standards and previously published isomeric profiles, as detailed by Nudda et al. [26].

The individual FAMEs were expressed as g/100 g of total FAME. The FA groups, expressed as g/100 g of total FAMEs or as g of FA/100 g of milk, were determined as follows: saturated fatty acids (SFAs) as a sum of the individual saturated FAs; unsaturated fatty acids (UFAs) as a sum of the individual unsaturated FAs; monounsaturated fatty acids (MUFAs) as a sum of the individual monounsaturated FAs; polyunsaturated fatty acids (PUFAs) as a sum of the individual polyunsaturated FAs; *anteiso* FA as a sum of individual *anteiso* FAs; PUFA n-3 as a sum of individual n-3 FAs; PUFA n-6 as a sum of individual n-6 FAs; total conjugated linoleic acid (CLA) as a sum of individual CLAs; short-chain fatty acids (SCFAs) as a sum of the individual FAs from C4:0 to C10:0; medium-chain fatty acids (MCFAs) as a sum of the individual FAs from C11:0 to C17:0; *trans* FA (TFAs) as a sum of individual trans FA; branched-chain FA (BCFAs) as a sum of individual branched-chain FAs; odd-chain FA (OCFAs) as a sum of individual odd-chain FAs; and odd- and branched-chain FAs (OBCFAs) as a sum of individual odd- and branched-chain FAs. De novo and preformed FAs, expressed as g FA/100 g of milk, were determined as described by Woolpert et al. [28]: de novo as a sum of FAs from C4:0 to C14:0, and preformed as a sum of FA ≥ C18:0.

The FA profile of the lyophilized herbage ryegrass samples was determined according to Kramer et al. [29], with the modification described by Correddu et al. [30]. The GC analysis of herbage samples was performed with the same equipment described above for the milk sample analysis. OpenLAB CDS GC ChemStation Upgrade software (Revision C.01.04, Agilent Technologies, Santa Clara, CA, USA) was used to calculate the retention time and area of each individual FAME in the herbage, which were then identified by comparing retention times with those of known standards and with published studies, as detailed by Nudda et al. [31].

Herbage samples were collected and analyzed concurrently with milk samples during the pre-experimental period and on experimental days 2, 5, 8 and 11. Body weight (BW) was measured using a precision scale (Biocontrol AS, Rakkestad, Norway). Body condition score (BCS) was assessed, using a scale ranging from 0 to 5, by the three trained individuals. Body weight and BCS were assessed on experimental days 1 and 9.

### 2.3. Statistical Analysis

Statistical analyses were performed using the statistical software Minitab 21.4.1 (Minitab, LLC, State College, PA, USA). The experimental unit was the ewe, since feed intake, milk yield, and milk composition were determined individually. Data on feed intake, milk yield and composition, BW and BCS were analyzed with a General Linear Model (GLM), with an experimental design based on analysis of variance (ANOVA), which included the fixed effects of treatment, day, and their interaction, and the random effect of animal. These data, except for the milk fatty acid profile, were covariate-adjusted using the mean of two pre-experimental measurements (2 and 5 April 2023). Data on milk fatty acid profile were analyzed with a GLM, with an experimental design based on ANOVA, which included the fixed effects of treatment and milking, and the random effect of animal.

Data were expressed as means and the group mean differences were assessed using the Tukey Pairwise Comparisons Test, with a significant *p*-value ≤ 0.05.

The statistical mixed model used for each variable (Y_ijk_), except for the milk fatty acid profile, was as follows:

Y_ijk_ = μ + treat_i_ + day_j_ + treat_i_ × day_j_ +anim_k_ + bx_ijk_ + error_ijk_

where μ is the mean, treat_i_ is the treatment effect (i = CNT; BIC), day_j_ is the experimental day of data collection (j = 2; 5; 8; 11), treat_i_ × day_j_ is the interaction, anim_k_ is the random effect of animal, b is the regression slope, x_ijk_ is the covariate (i.e., pre-experimental data), and error_ijk_ is the experimental error.

The statistical mixed model used for the milk fatty acid profile was as follows:

Y_ijk_ = μ + treat_i_ + milking_j_ + anim_k_ + error_ijk_

where μ is the mean, treat_i_ is the treatment effect (i = CNT; BIC), milking_j_ is the milking effect (j = morning; afternoon), anim_k_ is the random effect of animal, and error_ijk_ is the experimental error.

Exploratory Pearson correlation analysis was performed among the main dietary and production variables.

## 3. Results

### 3.1. Chemical Composition of Ryegrass Herbage

The chemical composition of the ryegrass herbage supplied to both groups of ewes (control and sodium bicarbonate) is reported in Table 2. The dry matter content had a mean of 22.7% and a maximum value of 25.5% of DM. The CP content was low, with a mean of 7.5% of DM and a minimum value of 6.7% of DM. In contrast, the WSC content was high, with an average value of 28% of DM and a maximum value of 29.8% of DM. The NFC content was also elevated, with a mean of 33.2% of DM and a maximum value of 35.6% of DM. The mean NDF content was 46.8% of DM, with a maximum value of 49% of DM. The mean ADF content was 27% of DM, and the mean ADL content was 5.7% of DM.

**Figure 2:**
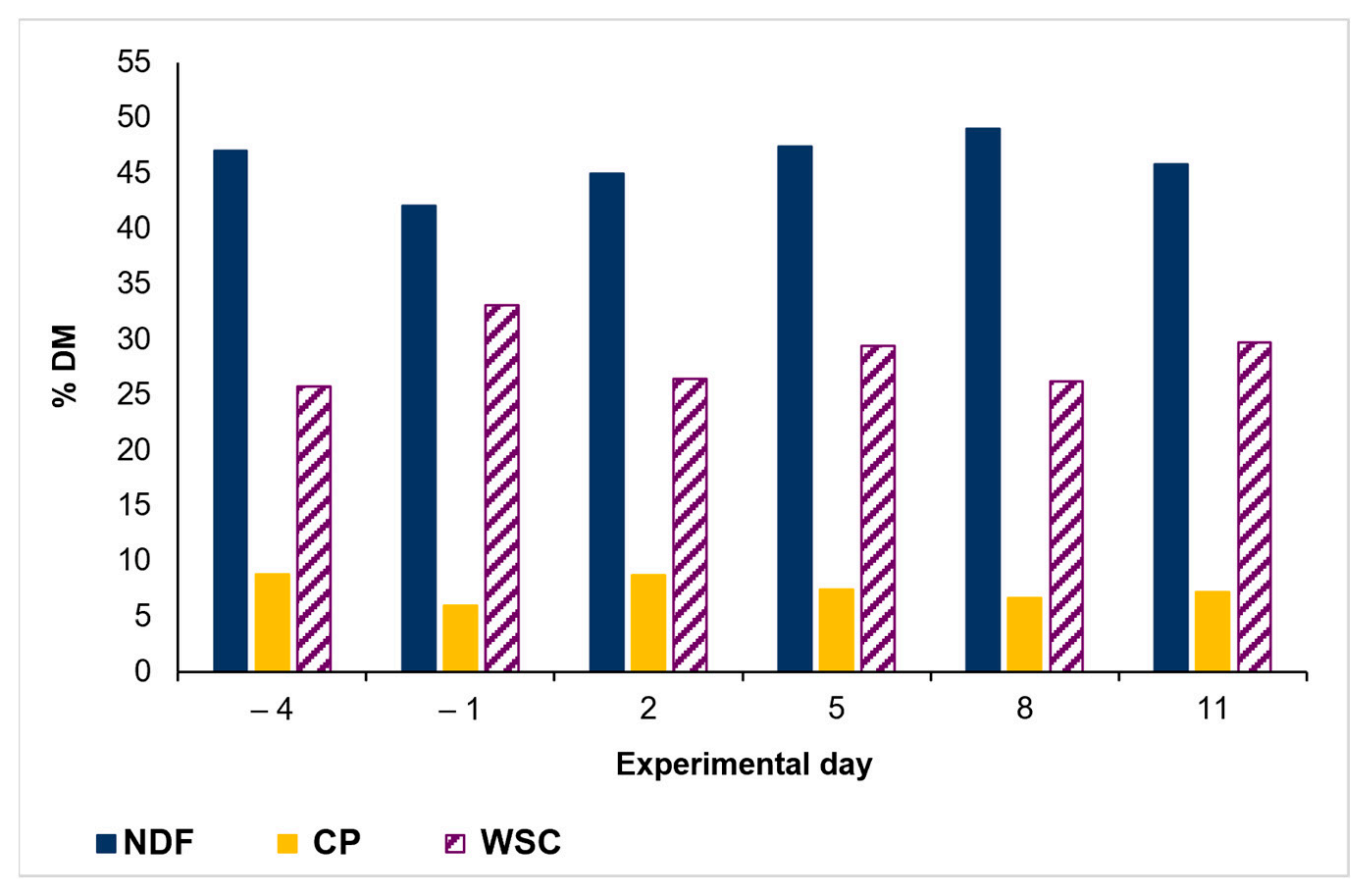
Chemical composition (neutral detergent fiber (NDF), crude protein (CP), and water-soluble carbohydrates (WSCs) of annual ryegrass herbage (*Lolium multiflorum* Lam.) supplied to the control (CNT, no sodium bicarbonate) and treated (BIC, sodium bicarbonate) ewe groups during the experimental period at various sampling dates.

Figure 2 reports the NDF, CP and WSC concentrations of the annual ryegrass herbage on the preliminary days −4 and −1 and on the experimental days 2, 5, 8 and 11 (corresponding to the milk sampling days), showing relatively constant concentrations throughout the experimental period. The grass WSC concentration was 25.8, 33.1, 26.5, 29.4, 26.2 and 29.8% of DM on preliminary days −4 and −1, and experimental days 2, 5, 8 and 11, respectively.

The fatty acid composition of ryegrass herbage reported in Table 2 shows that α-linolenic acid (C18:3 n-3) was the most abundant fatty acid, with a mean of 53.5 g/100 g of total FAME. The predominant fatty acids were C18:3 n-3, C16:0 (mean 20.1 g/100 g of total FAME) and C18:2 n-6 (mean 14.1 g/100 g of total FAME), which accounted for 87.7 g/100 g of total FAME.

### 3.2. Intake

Table 3 reports the covariate-adjusted means of dry matter and nutrient intake data for the control (CNT) and sodium-bicarbonate-treated (BIC) groups. Total DMI (g DM/d) was positively affected by sodium bicarbonate treatment (2319 vs. 2178 g DM/d for BIC and CNT, respectively; *p* = 0.03). The daily grass intake was significantly higher in BIC than in CNT (1488 vs. 1340 g DM/d, respectively; *p* = 0.03), whereas daily concentrate intake was not affected by treatment. The grass intake before the afternoon milking did not differ between the CNT and BIC groups, whereas it was higher in BIC than in CNT in the interval between the afternoon and subsequent morning milking (874 and 742 g DM/d, *p* = 0.02). The WSC intake was significantly higher in BIC than in CNT (418 and 377 g DM/d, *p* = 0.03), and it was higher in BIC than in CNT in the interval between the afternoon and subsequent morning milking (244 and 209 g DM/d, *p* = 0.02). Similarly, NDF intake was higher in BIC (857 vs. 791 g DM/d in BIC and CNT, respectively; *p* = 0.04). In contrast, CP intake did not differ between the two groups (Table 3).

**Figure 3:**
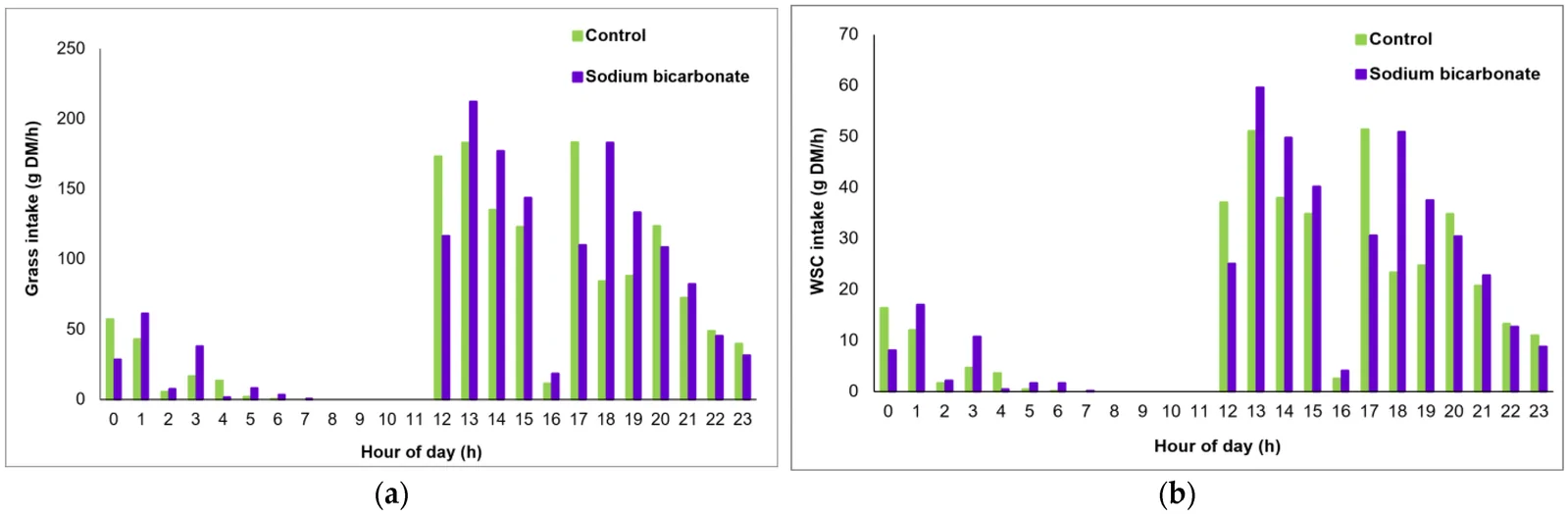
(**a**) Hourly annual ryegrass herbage (*Lolium multiflorum* Lam.) intake and (**b**) hourly water-soluble carbohydrate intake of the control (CNT, no sodium bicarbonate) and treated (BIC, sodium bicarbonate) ewe groups (average of experimental days 2, 5, 8, and 11) corresponding to milk sampling days.

Figure 3 reports the grass feeding behavior of the ewes as average hourly grass intake (g of DM/h; Figure 3a) and hourly WSC intake (g of DM/h; Figure 3b). Grass intake and, therefore, WSC intake before the afternoon milking (from 12:30 to 16:30) accounted for about 43% of total daily grass intake or total daily WSC intake in both groups, and during the nighttime hours (from midnight to 07:30), before the morning milking, accounted for about 10% of the total daily grass intake or total daily WSC intake in both groups.

### 3.3. Body Weight, Body Condition Score, Milk Yield and Milk Chemical Composition

The covariate-adjusted mean data on BW, BCS, milk yield, and milk composition for the CNT and BIC groups are reported in Table 4.

The BW was not affected by the treatment, with mean values of 57.8 and 58.2 kg in the CNT and BIC groups, respectively. Likewise, BCS did not differ between CNT and BIC, with mean values of 3.1 and 2.9, respectively (Table 4).

Daily milk yield was not affected by treatment, with mean values of 1859 and 1838 g/d in CNT and BIC, respectively. Additionally, there were no differences in the afternoon and morning milk yields between the two groups either. On the other hand, daily milk fat concentration was significantly higher in BIC than in CNT, with values of 5.79 and 5.41%, respectively (*p* < 0.01). Furthermore, BIC also had significantly higher values than CNT regarding milk fat concentration in the afternoon milking (6.52 vs. 6.01% in BIC and CNT, respectively; *p* < 0.001) and in the subsequent morning milking (5.39 and 5.06%, in BIC and CNT, respectively; *p* = 0.03) (Table 4).

**Figure 4:**
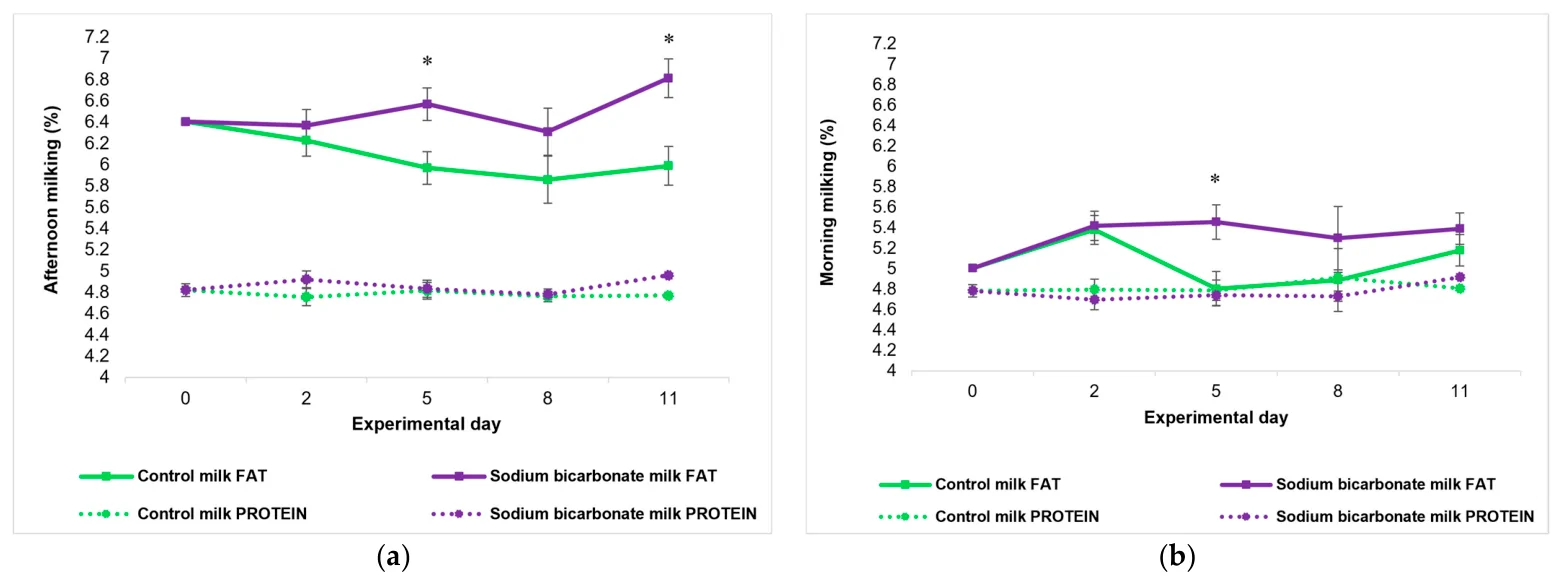
Covariate-adjusted means and standard error of the mean (SEM) of milk fat (solid line) and protein (dashed line) concentration (%) in the (**a**) afternoon milking and (**b**) subsequent morning milking of the control (CNT, no sodium bicarbonate) and treated (BIC, sodium bicarbonate) ewe groups at various sampling dates. Means with * within the same experimental day differ (*p* ≤ 0.05).

Figure 4 shows the covariate-adjusted means over time of milk fat concentration in the afternoon (Figure 4a) and subsequent morning milkings (Figure 4b) during the experimental period. Milk fat concentration in the afternoon milking (Figure 4a) was higher in BIC group than in CNT over time, although the difference was statistically significant only on experimental days 5 and 11. The milk fat concentration in the morning milking (Figure 4b) had higher values in BIC than in CNT on experimental days 5, 8 and 11, but showing a statistical significance only on experimental day 5.

Daily fat yield was significantly higher in BIC than in CNT (106.5 vs. 99.7 g/milking; *p* = 0.05; Table 4). The fat yield in the afternoon milking was also significantly higher in BIC than in CNT (42.2 vs. 38.7 g/milking; *p* = 0.03). However, the fat yield in the morning milking was not affected by sodium bicarbonate (*p* = 0.18). The daily and morning milk protein concentrations were not influenced by sodium bicarbonate, whereas the afternoon milk protein concentration tended to be higher in BIC than in CNT (*p* = 0.07) (Table 4).

Figure 4 also shows the covariate-adjusted mean of milk protein concentration over time in the afternoon (Figure 4a) and subsequent morning milkings (Figure 4b), with no differences between the CNT and BIC groups throughout the experimental period.

The daily milk fat to protein ratio was significantly influenced by treatment, being higher in BIC than in CNT (1.21 vs. 1.13; *p* < 0.001). The milk fat to protein ratio was also higher in BIC than in CNT in the afternoon milking (1.35 vs. 1.25; *p* < 0.001) and in the morning milking (1.13 vs. 1.06; *p* = 0.01) (Table 4).

Table 5 shows the milk fatty acid profile, expressed as g/100 g of total FAME, of the CNT and BIC ewe groups on the last day of the animal experiment (day 12).

The CNT group showed significantly higher values than BIC regarding C17:0 FA (*p* = 0.04), the sum of C17:0 and C17:1 *cis* 9 (*p* = 0.04), and *anteiso* C17:0 (*p* < 0.01), whereas C17:1 *cis* 9 tended to be higher in CNT than in BIC (*p* = 0.07) (Table 5). Linoleic acid (C18:2 n-6) (*p* = 0.02) and linolenic acid (C18:3 n-3) (*p* < 0.01) were significantly higher in CNT than in BIC. On the other hand, C18:1 *trans* 6–8 was higher in BIC than in CNT (*p* = 0.04). Docosapentaenoic acid (DPA; C22:5 n-3) was higher in CNT than in BIC (*p* < 0.01) and eicosapentaenoic acid (EPA; C20:5 n-3) tended to be higher in CNT than in BIC (*p* = 0.07). Total PUFA was significantly higher in CNT than in BIC (*p* < 0.01). Additionally, PUFA n-3 (*p* < 0.001) and PUFA n-6 (*p* = 0.01) were also significantly higher in CNT than in BIC. In contrast, the n-6/n-3 ratio was significantly higher in BIC than in CNT (*p* = 0.03). The CNT had significantly higher content than BIC regarding OCFA (*p* = 0.03), BCFA (*p* < 0.01) and OBCFA (*p* < 0.001). The *anteiso* FAs were also higher in CNT than in BIC (*p* < 0.01) (Table 5). The complete milk fatty acid profile, expressed as g/100 g of FAME, of the CNT and BIC groups on the last day of animal experiment (day 12), is reported in Table S1.

Table 6 reports the main milk fatty acid groups, expressed as g FA/100 g of milk, of the CNT and BIC groups on the last day of animal experiment (day 12). The BCFAs, OCFAs and OBCFAs were not affected by treatment, whereas the SFA tended to be higher in BIC than in CNT (*p* = 0.08). SCFAs and MCFAs did not differ between CNT and BIC, but the de novo fatty acids tended to be higher in BIC than in CNT (*p* = 0.09). Conversely, the preformed fatty acids (LCFAs) were not affected by sodium bicarbonate. The n-6/n-3 ratio was significantly higher in BIC than in CNT (*p* = 0.03).

## 4. Discussion

### 4.1. Chemical Composition of Ryegrass Herbage

The high WSC concentration (28% of DM) of the fresh annual ryegrass used in our study is consistent with that of King et al. [32], who reported that Italian ryegrass (*Lolium multiflorum* Lam.) is capable of accumulating high concentrations of WSC. Similarly, Rivero et al. [1] reported average WSC concentrations of 33.2% DM in high-sugar perennial ryegrass cultivars grown in south-central Chile, a region with a temperate Mediterranean climate. Although such high WSC concentrations have been considered typical of grasses grown in high-latitude regions [4], recent climatic changes in Mediterranean environments, including a higher difference between day and night temperatures and more frequent winter dry spells, may also favor WSC accumulation [6,7]. In addition, the high WSC concentration can also be attributed to the tetraploid ryegrass cultivar used in the present study, because tetraploid cultivars generally accumulate more WSCs than diploid cultivars [33,34], and to the observed cool, sunny, and relatively dry conditions (Table 1) which favor WSC accumulation [33,35,36].

The observed low CP concentration (7.5% of DM) of the herbage used was consistent with previous studies [1,3] reporting a negative correlation between WSC and CP concentrations in herbage.

The chemical composition of the ryegrass herbage used in the present study likely resulted from the combined effects of species and variety and environmental conditions. The fatty acid composition of the annual ryegrass herbage used in the trial was comparable with values reported for annual ryegrass in other studies [37,38], with linolenic acid (C18:3 n-3), oleic acid (C16:0) and linoleic acid (C18:2 n-6) being the most represented fatty acids.

### 4.2. Effect of Sodium Bicarbonate (NaHCO_3_) Supplementation on Intake, Milk Yield, and Milk Composition

The positive effects of sodium bicarbonate supplementation at 25 g/day (about 1% of total DMI) on total DMI, with higher values for the supplemented group, are consistent with the study of Vicini et al. [39], who found that supplementation with sodium bicarbonate at doses of 1% of DM increased the DMI of roughage and total DMI in dairy cows fed a diet consisting of corn silage (50% of DM) and concentrates (50% of DM) ad libitum.

Because the daily grass intake was significantly higher in BIC than in CNT and the daily concentrate intake was not affected by sodium bicarbonate, the observed higher total DMI in BIC can be attributed to the higher grass intake in that group. Similarly, Wittayakun et al. [40] reported that the addition of sodium bicarbonate (1.2% of DM) to the diet of dairy cows fed forages and concentrates positively affected roughage intake, although it did not affect total intake. In our study, the significantly higher WSC and NDF intake in the BIC group, compared to the CNT group, reflected the greater grass intake in BIC, whereas the lack of difference in CP intake between the groups can be attributed to the high contribution of soybean meal to total dietary protein. The higher herbage intake may be related to a greater dilution rate of rumen fluid caused by sodium bicarbonate supplementation, which is known to reduce the retention time of DM [18], or by a higher rumen pH, which favors fiber digestibility [41]. In fact, Pérez-Ruchel et al. [16] reported that 20 g/d of a combination of sodium bicarbonate and magnesium oxide (75% and 25%), supplied to wethers fed fresh herbage indoors, increased ruminal fluid outflow rate and rumen pH, as early as 4 h after buffer supplementation, with these effects persisting for up to 24 h thereafter. Therefore, these previously reported effects might have caused the significantly higher grass intake in BIC than in CNT, and consequently higher WSC intake, in the interval from the afternoon milking (16:30) to the morning milking (07:30), following sodium bicarbonate supplementation just before the start of grass supply (12:30 h).

The observed lack of effect of sodium bicarbonate on daily, afternoon and morning milk yields is consistent with Wittayakun et al. [40], who observed no effect of 1.2% sodium bicarbonate on milk yield in dairy cows fed pineapple peel and commercial pellet (ratio of 70:30), despite a higher DMI in the supplemented group, as also found in the present study. In contrast, Cruywagen et al. [23] reported that sodium bicarbonate supplementation (0.8% of DM) increased milk yield in dairy cows fed a total mixed ration with low forage content (35.2% forage on total DM). These discrepancies could be attributed to the fact that in some studies maximum milk yield had already been reached and the extra nutrient intake allowed by the use of the buffer was partitioned in favor of milk components or body reserves.

Despite the significantly higher daily WSC intake in the BIC group, which was negatively correlated with milk fat concentration in other studies [3,11], the daily milk fat concentration in the BIC group was higher compared to the CNT group. In particular, a strong positive effect of sodium bicarbonate on milk fat concentration was observed in the afternoon milking (6.52 vs. 6.01% for BIC and CNT, respectively; *p* < 0.01), which occurred four hours after supplying the buffer. Indeed, the afternoon milk fat concentration was negatively associated with WSC intake (r = −0.44; *p* = 0.05) in the CNT group but positively associated with WSC intake (r = +0.46; *p* = 0.04) in the BIC group. Although NDF intake was significantly higher in BIC than in CNT, due to the higher herbage intake in the treated group, it was not associated with afternoon milk fat concentration in BIC (r = 0.29; *p* = 0.21). The fact that milk fat concentration in each milking was significantly higher in the BIC group, even though milk yield did not differ in any milking between the groups, indicates that this is not a milk composition dilution-related effect. These findings are consistent with those of Cruywagen et al. [23], who reported an increase in milk fat concentration in dairy cows fed a high-concentrate total mixed ration (forage: 35.2% of DM) supplemented with sodium bicarbonate at 0.8% of DM, and with those of Hadjipanayiotou [20], who observed a similar increase in milk fat concentration in dairy goats fed hay and concentrates supplemented with sodium bicarbonate at 4% of concentrate (ca. 2.8–3.4% of DM).

The positive response of milk fat concentration to sodium bicarbonate used at the dose of 1% of DM, observed in our study, may also be due to the fact that the ability of dietary buffers to increase ruminal pH is greater in low-forage diets compared to high-forage diets [12] and is more pronounced at lower rumen pH [13,14,19]. In our study, animals were fed exclusively ryegrass herbage rich in WSCs (28% of DM) ad libitum and rationed concentrates, and the forage component of the diet consisted entirely of fresh grass, characterized by lower concentration of NDF, and likely of peNDF, and higher moisture content compared to the forage used in previous studies. It is important to highlight that in a meta-analysis of early- and mid-lactation dairy cows, Hu and Murphy [14] described a positive impact of sodium bicarbonate on DMI and productivity in cows fed solely or predominantly maize silage-based diets, probably for the high starch content of this type of forage but found no impact on cows fed other forage sources. The authors stated that responses of ruminants to sodium bicarbonate vary with the diet type, especially with the forage used, and may be related to differences in the fiber concentration of the forage.

The milk protein concentration was not affected by sodium bicarbonate, but there was a strong trend in the afternoon milking, as reported previously [20]. The higher milk fat to protein ratio in the BIC group at each milking, especially in the afternoon milking, can be attributed to a higher milk fat concentration. Although the milk fat to protein ratio at each milking was significantly higher in BIC than in CNT, this ratio remained above the inversion threshold of 1 [42,43] in both groups. A low milk fat to protein ratio (<1) is correlated with low rumen pH and is considered an indicator of subacute ruminal acidosis (SARA) [42,44]. In our study the fat to protein ratio remained positive and high, likely due to the low milk protein concentration observed in both groups (Figure 4a,b), even at the low fat concentration values of the CNT group. In fact, during ruminal acidosis, the milk fat content decreases, but the milk protein content can also decrease [45,46] because ruminal microbial protein synthesis is altered [45], thus resulting in a reduced supply of precursors for protein synthesis in the mammary gland.

The positive effect of sodium bicarbonate supplementation on milk fat, with increased milk fat concentration in both milkings, but marked increases in the afternoon as well as increased milk fat yield only in the afternoon milking, i.e., four hours after sodium bicarbonate supply, indicates a marked rapid effect of this buffer. The observed rapid effect of sodium bicarbonate could be explained by its rapid buffering action at the ruminal level, which prevents post-prandial increases in H^+^ [17]. Additionally, it is important to remember that in the hours before the afternoon milking (from 12:30 to 16:30), grass intake accounted for 42.7% and 43% of the total daily grass intake (g DM/d) in CNT and BIC, respectively, and that grass intake during the nighttime hours (from midnight to 07:30), before morning milking, was very low (about 10%; Figure 3a). This means that about 43% of the daily WSC intake was also consumed in four hours, whereas the rest of the WSC were ingested over the subsequent 14 h (from 17:00 to 07:30 of the subsequent day; Figure 3b). Molle et al. [2] reported a negative correlation (r = −0.57) between pasture WSC intake from grasses and ruminal pH in grazing dairy ewes, and thus ruminal pH was likely lower in the hours preceding the afternoon milking than before the morning milking in our trial. This could explain the marked effect observed in the afternoon milking, considering that the response to sodium bicarbonate is influenced by the diet and is greater at low ruminal pH [13,14,19].

With regard to the milk fatty acid profile, the higher concentrations of C17:0, *anteiso* C17:0, and the sum of C17:0 and C17:1 *cis* 9, as well as a tendency to higher C17:1 *cis* 9 concentration, observed in CNT compared to BIC, may be associated with the enrichment of OCFAs [47,48,49] in rumen amylolytic bacteria, consistent with the significantly higher OCFAs concentrations in CNT group. Indeed, C17:0 is positively correlated with the proportion of propionate in the rumen, as C17:0 increases as the amount of concentrate in the diet increases [47,50,51]. Additionally, *anteiso* C17:0 is an indicator of the consumption of high-starch diets, because this type of diet can lead to a more active elongation of *anteiso* C15:0 to *anteiso* C17:0 [49,50]. Higher milk proportions of C15:0, C17:0 and/or C17:1 *cis* 9 (and their sum), and *anteiso* FAs have been reported as biomarkers and indicators of ruminal acidosis, with elevated levels of C17:0 also indicating protein deficiency [48,52]. In our study, we observed higher *anteiso* FAs (*p* < 0.01) in CNT than in BIC. The higher levels of PUFA, PUFA n-3, and PUFA n-6 observed in the CNT group are likely due to a higher rate of rumen biohydrogenation in the BIC group, resulting in lower concentrations of polyunsaturated FAs in the milk of supplemented ewes. Moreover, the same mechanism may explain the higher C18:3 n-3 in CNT, despite the greater intake of grass in BIC. In fact, high-forage diets generally tend to produce milk with a greater content of C18:3 n-3 [12]. In addition, the higher C18:2 n-6 concentration detected in CNT likely led to increased PUFA n-6 concentrations, and this FA is typically elevated in milk from ruminants fed high-concentrate diets and/or low rumen pH conditions [48,53,54]. Differently, the de novo fatty acids (g/100 g milk), which tended to be higher in BIC, are positively associated with milk fat and protein content [28] and are an indicator of intense ruminal fermentation conditions [28], being synthesized in the mammary gland from acetate and butyrate of ruminal origin. De novo FA are strongly related to nutrition and may be depressed by high dietary quickly fermented carbohydrates and PUFA [55].

We hypothesize that the positive effect of sodium bicarbonate on milk fat concentration and yield might also be a consequence of an increased water intake and saliva production, often caused by the use of buffer salts, which leads to a subsequent rise in the rumen liquid dilution rate [17] and consequent increase in the outflow of fermentable carbohydrates from the rumen, thus resulting in an increase in ruminal pH and, consequently, higher milk fat production [13,17]. The increased ruminal pH resulting from sodium bicarbonate supplementation has been well documented [18,19,22] although it was not observed in a few studies [20,40].

In our study, we chose to use the rumen buffer sodium bicarbonate due to its rapid buffering action at the ruminal level, because WSCs are commonly considered a group of rapidly degraded carbohydrates by ruminal microbes. WSCs have a fermentation rate of up to 300%/h [56], although this rate likely differs between different types of WSCs, depending on factors such as their degree of polymerization (DP) [57,58]. Combining rapid-acting ruminal buffers like sodium bicarbonate with alkalizers such as magnesium oxide (MgO), which have a slower action (effective after 24 h) and a postabsorptive effect rather than a ruminal effect [13], could potentially have an additive effect on milk fat concentration in pasture-fed ewes, resulting in longer-lasting effects. Additionally, providing hay with high NDF and peNDF content before grazing WSC-rich grasses can be beneficial by increasing chewing time, rumination, salivation, thereby exerting a buffering action in the rumen. However, it is important to consider that this trial was conducted indoors, and the feeding behavior of grazing ewes may differ depending on plant species, plant part selection, grass intake rate, and access time to pasture. Thus, total and hourly intake of WSC during grazing under field conditions and the negative effects of WSC excess could be exacerbated in grazing ewes compared to those observed indoors.

## 5. Conclusions

The inclusion of sodium bicarbonate, supplied as a ruminal buffer just before the herbage supply, in the diet of ewes fed ryegrass herbage rich in WSC (28% of DM) increased total dry matter intake, herbage intake, milk fat concentration, and milk fat yield. The increase in milk fat concentration and milk fat yield, observed especially in the afternoon milking, was likely due to the rapid effect of this buffer on the rumen. Thus, sodium bicarbonate may increase milk fat concentration in grazing ewes consuming pastures rich in WSC and low in CP, provided that it is supplied prior to grazing, given its rapid effect on the rumen. Finally, it could be useful to compare the use of sodium bicarbonate alone with its combination with slow action alkalinizes that could extend the buffering effect over time, such as magnesium oxide, both indoors and under field conditions.

## Figures and Tables

**Table 1:** Monthly mean weather values for minimum and maximum temperatures, solar radiation and total rainfall at the experimental farm of the Department of Agricultural Sciences of the University of Sassari (Ottava, Sassari, Italy) from annual ryegrass sowing (18 October 2022) to the end of the trial (17 April 2023). The 12-day animal experiment lasted from 6 to 17 April 2023.

	Months						
Item	October	November	December	January	February	March	April
Mean minimum temperature (°C)	15.5	12.3	10.4	7.8	6.1	8.8	7.6
Mean maximum temperature (°C)	26	20.4	18.7	14.8	15.3	18.3	17.3
Mean solar radiation (MJ/m^2^ per day)	14	8.7	7.9	7.9	11.3	16.2	20.4
Rainfall, sum (mm/month)	15.6	200.3	113.9	53.2	13.6	6.6	4.6

**Table 2:** Chemical composition of freshly cut ryegrass herbage (*n* = 4) during the animal experiment (average of sampling days 2, 5, 8 and 11). The 12-day animal experiment lasted from 6 to 17 April 2023.

Item ^1^ (% of DM, Unless Stated Otherwise)	Mean	Minimum	Maximum
Dry matter (% of fresh matter)	22.7	21.6	25.5
Crude protein	7.5	6.7	8.7
Soluble crude protein	3.9	3.4	4.5
Acid detergent insoluble crude protein	0.66	0.61	0.76
Ether extract	1.9	1.7	2.1
Ash	9.8	8.9	11.3
Neutral detergent fiber	46.8	45	49
Acid detergent fiber	27	24.7	29.4
Acid detergent lignin	5.7	5	6.3
Non-fiber carbohydrates	33.2	29.7	35.6
Water-soluble carbohydrates	28	26.2	29.8
**Fatty Acid Profile (g/100 g of Total FAME)**			
C12:0	0.66	0.51	0.81
C14:0	0.70	0.45	1.03
C15:0	0.17	0.13	0.26
C16:0	20.1	18.3	25.7
C16:1 *cis*-7	1.53	1.45	1.67
C16:1 *cis*-9	0.51	0.34	0.73
C17:0	0.28	0.21	0.39
C17:1 *cis*-10	0.12	0.11	0.15
C18:0	2.03	1.39	2.58
C18:1 *cis*-9	4.08	2.37	6.55
C18:2 n-6	14.1	13.1	15.3
C20:0	0.50	0.37	0.73
C18:3 n-3	53.5	43.4	60.2
C20:3 n-3 (10, 14, 17)	0.05	0.04	0.05
C24:0	0.85	0.69	1.04

^1^ All feed components were analyzed by NIRS, except for WSCs, which were analyzed using a chemical method (anthrone sulphate colorimetric method), and fatty acids, which were analyzed by gas-chromatography.

**Table 3:** Covariate-adjusted means of daily dry matter and nutrient intake of the control (CNT, no sodium bicarbonate) and treated (BIC, sodium bicarbonate) ewe groups during the animal experiment (from 6 to 17 April 2023).

Item	Treatment		SEM	*p*-Value
	CNT	BIC		
Total dry matter intake (g DM/d)	2178	2319	44.2	0.03
Daily grass intake (g DM/d)	1340	1488	45.9	0.03
Daily concentrate intake (g DM/d)	843	826	17	0.50
Grass intake, afternoon ^1^ (g DM/d)	592	620	36	0.6
Grass intake, between afternoon and morning milking ^2^ (g DM/d)	742	874	35.5	0.02
Water-soluble carbohydrate intake (g DM/d)	377	418	12.8	0.03
Water-soluble carbohydrate intake, afternoon ^1^ (g DM/d)	167	175	9.8	0.57
Water-soluble carbohydrate intake, between afternoon and morning milking ^2^ (g DM/d)	209	244	9.85	0.02
Neutral detergent fiber intake (g DM/d)	791	857	21.8	0.04
Crude protein (g DM/d)	331	335	6.28	0.64

^1^ Afternoon = from 12:30 to 16:30 (before the 16:30 afternoon milking); ^2^ between afternoon and morning milking = from 17:00 to 07:30 (morning milking).

**Table 4:** Covariate-adjusted means of body weight, body condition score, milk yield and composition of the control (CNT, no sodium bicarbonate) and treated (BIC, sodium bicarbonate) ewe groups.

Item	Treatment		SEM	*p*-Value
	CNT	BIC		
Body weight (kg)	57.8	58.2	0.34	0.41
Body condition score (1–5 points)	3.1	2.9	0.09	0.20
Milk yield (g/d)	1859	1838	28.6	0.62
Milk yield, afternoon ^1^ (g/milking)	649	647	16.1	0.91
Milk yield, morning ^2^ (g/milking)	1206	1194	24.5	0.74
Milk fat concentration (%)	5.41	5.79	0.08	<0.01
Milk fat concentration, afternoon ^1^ (%)	6.01	6.52	0.09	<0.001
Milk fat concentration, morning ^2^ (%)	5.06	5.39	0.1	0.03
Fat yield (g/d)	99.7	106.5	2.34	0.05
Fat yield, afternoon ^1^ (g/milking)	38.7	42.2	1.12	0.03
Fat yield, morning ^2^ (g/milking)	60.6	64.7	2.03	0.18
Milk protein concentration (%)	4.81	4.81	0.04	0.92
Milk protein concentration, afternoon ^1^ (%)	4.78	4.87	0.03	0.07
Milk protein concentration, morning ^2^ (%)	4.83	4.77	0.04	0.34
Milk fat to protein ratio	1.13	1.21	0.01	<0.001
Milk fat to protein ratio, afternoon ^1^	1.25	1.35	0.02	<0.001
Milk fat to protein ratio, morning ^2^	1.06	1.13	0.02	0.01

^1^ Afternoon = milk from the 16:30 milking; ^2^ morning = milk from the 07:30 milking of the subsequent day.

**Table 5:** Milk fatty acid profile (g/100 g of total fatty acid methyl esters (FAME)) of the control (CNT, no sodium bicarbonate) and treated (BIC, sodium bicarbonate) ewe groups on the last day of the animal experiment (day 12).

Item	Treatment		SEM	*p*-Value
	CNT	BIC		
C4:0	2.37	2.39	0.04	0.74
C6:0	2.23	2.22	0.06	0.86
C8:0	2.30	2.30	0.10	0.95
C10:0	7.46	7.88	0.333	0.39
C12:0	4.29	4.59	0.187	0.28
*iso* C13:0	0.03	0.02	0.002	0.88
C14:0	11.4	11.2	0.21	0.43
*iso* C14:0	0.16	0.14	0.01	0.22
C15:0	1.17	1.09	0.04	0.16
*iso* C15:0	0.35	0.33	0.01	0.28
C16:0	25.3	26	0.63	0.44
C16:1 *cis* 9	0.78	0.82	0.04	0.49
*iso* C16:0	0.37	0.37	0.01	0.71
C17:0	0.90	0.82	0.02	0.04
C17:1 *cis* 9	0.29	0.25	0.01	0.07
C17:0 + C17:1 *cis* 9	1.19	1.07	0.04	0.04
C17:1 *cis* 9/C17:0	0.32	0.30	0.01	0.23
*anteiso* C17:0	0.51	0.45	0.01	<0.01
C18:0	9.55	10.1	0.25	0.10
C18:1 *trans* 6–8	0.19	0.21	0.007	0.04
C18:1 *trans* 9	0.17	0.18	0.006	0.26
C18:1 *trans* 10	0.28	0.30	0.02	0.48
C18:1 *trans* 11	0.80	0.80	0.04	0.97
C18:1 *trans* 12	0.23	0.25	0.01	0.24
C18:1 *cis* 9	19.6	18.7	0.69	0.27
C18:1 *cis* 11	0.38	0.36	0.01	0.23
C18:1 *cis* 12	0.16	0.17	0.008	0.54
CLA C18:2 *trans* 10, *cis* 12	0.007	0.008	0.0007	0.67
C18:2 n-6	2.18	1.90	0.07	0.02
C18:3 n-3	0.77	0.59	0.02	<0.01
CLA *cis* 9, *trans* 11	0.45	0.43	0.02	0.33
C20:5 n-3	0.07	0.06	0.003	0.07
C22:5 n-3	0.11	0.09	0.003	<0.01
C22:6 n-3	0.02	0.02	0.002	0.52
SFA	70.4	71.9	0.64	0.12
UFA	30.1	28.7	0.61	0.11
MUFA	25.5	24.5	0.59	0.28
PUFA	4.64	4.14	0.09	<0.01
PUFA n-3	0.87	0.69	0.02	<0.001
PUFA n-6	2.72	2.40	0.08	0.01
n-6/n-3 ratio	3.12	3.48	0.11	0.03
Total CLA	0.62	0.61	0.02	0.68
*anteiso* FA	1.07	0.98	0.02	<0.01
OCFA	2.77	2.60	0.05	0.03
BCFA	2.08	1.95	0.03	<0.01
OBCFA	3.68	3.46	0.03	<0.001

SFAs = saturated fatty acids; UFAs = unsaturated fatty acids; MUFAs = monounsaturated fatty acids; PUFAs = polyunsaturated fatty acids; OCFAs = sum of individual odd-chain fatty acids; BCFAs = sum of individual branched-chain fatty acids; OBCFAs = sum of individual odd- and branched-chain fatty acids.

**Table 6:** Milk fatty acids groups (g fatty acid/100 g milk) of the control (CNT, no sodium bicarbonate) and treated (BIC, sodium bicarbonate) ewe groups on the last day of the animal experiment (day 12).

Item	Treatment		SEM	*p*-Value
	CNT	BIC		
SCFA	0.68	0.78	0.04	0.10
MCFA	1.59	1.82	0.10	0.12
De novo fatty acids	1.22	1.39	0.07	0.09
Preformed fatty acids (LCFAs)	1.32	1.46	0.08	0.28
De novo/preformed fatty acids ratio	0.95	0.97	0.03	0.69
BCFA	0.07	0.07	0.004	0.42
OCFA	0.10	0.10	0.005	0.41
OBCFA	0.17	0.18	0.009	0.41
TFA	0.14	0.16	0.01	0.13
SFA	2.47	2.86	0.14	0.08
UFA	1.05	1.12	0.07	0.47
MUFA	0.88	0.95	0.06	0.42
PUFA	0.17	0.17	0.01	0.89
PUFA n-3	0.03	0.03	0.001	0.13
PUFA n-6	0.09	0.09	0.01	0.92
n-6/n-3 ratio	3.12	3.49	0.11	0.03
Total CLA	0.02	0.02	0.001	0.22

SCFAs = short-chain fatty acids, from C4:0 to C10:0; MCFAs = medium-chain fatty acids, from C11:0 to C17:0; de novo = from C4:0 to C14:0; preformed = ≥C18:0; BCFAs = sum of individual branched-chain fatty acids; OCFAs = sum of individual odd-chain fatty acids; OBCFAs = sum of individual odd- and branched-chain fatty acids; TFAs = sum of individual trans fatty acids; SFAs = saturated fatty acids; UFAs = unsaturated fatty acids; MUFAs = monounsaturated fatty acids; PUFAs = polyunsaturated fatty acids.

## Data Availability

All data generated or analyzed during this study are included in the article/Supplementary Materials. Further information is available from the corresponding author.

## Supplementary Materials

The supplementary data presented in the study are openly available at https://www.mdpi.com/article/10.3390/ani16162481/s1, Table S1. Complete milk fatty acid profile (g/100 g of total fatty acid methyl esters (FAME)) of the control (CNT, no sodium bicarbonate) and treated (BIC, sodium bicarbonate) ewe groups on the last day of the animal experiment (day 12).

## Abbreviations

The following abbreviations are used in this manuscript:

ADF	Acid detergent fiber
ADICP	Acid detergent insoluble crude protein
ADL	Acid detergent lignin
ANOVA	Analysis of variance
BCFA	Branched-chain fatty acids
BCS	Body condition score
BIC	Sodium bicarbonate group
BW	Body weight
CLA	Conjugated linoleic acid
CNT	Control group
CP	Crude protein
DHA	Docosahexaenoic acid
DM	Dry matter
DMI	Dry matter intake
DP	Degree of polymerization
DPA	Docosapentaenoic acid
EE	Ether extract
EPA	Eicosapentaenoic acid
FA	Fatty acids
FAME	Fatty acid methyl esters
FID	flame ionization detector
GC	Gas chromatography
GLM	General Linear Model
MCFA	Medium-chain fatty acids
MFD	Milk fat depression
MUFA	Monounsaturated fatty acids
NDF	Neutral detergent fiber
NIRS	Near-infrared reflectance spectroscopy
OBCFA	Odd- and branched-chain fatty acids
OCFA	Odd-chain fatty acids
PUFA	Polyunsaturated fatty acids
SARA	Subacute ruminal acidosis
SD	Standard deviation
SFA	Saturated fatty acids
SCFA	Short-chain fatty acids
TFA	Trans fatty acids
UFA	Unsaturated fatty acids
VFA	Volatile fatty acids
WSC	Water-soluble carbohydrates
