# Supplementary material for: Sodium Bicarbonate Can Increase Milk Fat Concentration in Lactating Ewes Fed Ryegrass Herbage Rich in Water-Soluble Carbohydrates
Source: Animals (Basel). 2026 Aug 10;16(16):2481. doi: 10.3390/ani16162481 (PMC13508939; doi:10.3390/ani16162481)
Supplement: Supplementary file 1 [file animals-16-02481-s001.zip › animals-4482839-supplementary.pdf]

Sodium Bicarbonate Can Increase Milk Fat Concentration in Lactating Ewes Fed Ryegrass Herbage Rich in Water-Soluble Carbohydrates

Maria Angela Porcu *, Antonello Ledda, Silvia Carta, Ana Helena Dias Francesconi and Antonello Cannas

Department of Agricultural Sciences, University of Sassari, 07100 Sassari, Italy; anledda@uniss.it (A.L.); scarta2@uniss.it (S.C.); france@uniss.it (A.H.D.F.); cannas@uniss.it (A.C.)
* Correspondence: maporcu1@uniss.it

Supplementary Table S1.

Complete milk fatty acid profile (g/100 g of total fatty acid methyl esters (FAME)) of the control (CNT, no sodium bicarbonate) and treated (BIC, sodium bicarbonate) ewe groups on the last day of the animal experiment (day 12).

Item	Treatment		SEM	*p*-value
	CNT	BIC		
C4:0	2.37	2.39	0.04	0.74
C6:0	2.23	2.22	0.06	0.86
C7:0	0.02	0.02	0.002	0.50
C8:0	2.30	2.30	0.10	0.95
C9:0	0.23	0.22	0.006	0.46
C10:0	7.46	7.88	0.333	0.39
C10:1	0.01	0.01	0.0003	0.14
C11:0	0.37	0.36	0.02	0.79
C12:0	4.29	4.59	0.187	0.28
iso C13:0	0.03	0.02	0.002	0.88
*anteis*o C13:0	0.05	0.05	0.003	0.69
iso C14:0	0.16	0.14	0.01	0.22
C14:0	11.4	11.2	0.21	0.43
iso C15:0	0.35	0.33	0.01	0.28
anteiso C15:0	0.51	0.48	0.02	0.29
C14:1 *cis* 9	0.19	0.18	0.01	0.53
C15:0	1.17	1.09	0.04	0.16
C15:1 *cis* 9	0.06	0.06	0.002	0.45

iso C16:0	0.37	0.37	0.01	0.71
C16:0	25.3	26	0.63	0.44
C16:1 *trans* 4	0.01	0.01	0.002	0.06
C16:1 *trans* 5	0.03	0.01	0.005	0.07
C16:1 *trans* 6-7	0.04	0.02	0.006	0.13
iso C17:0	0.04	0.04	0.002	0.34
C16:1 *trans* 9	0.58[a]	0.53[b]	0.01	0.02
C16:1 *trans* 10	0.01	0.01	0.001	0.68
C16:1 *trans* 11- *trans* 12	0.05	0.05	0.002	0.69
C16:1 *cis* 7	0.31	0.32	0.01	0.87
anteiso C17:0	0.51[a]	0.45[b]	0.01	<0.01
C16:1 *cis* 9	0.78	0.82	0.04	0.49
C16:1 *cis* 10	0.03	0.02	0.002	0.31
C16:1 *cis* 11	0.01	0.01	0.001	0.70
3,7,11,15-Tetramethyl-16:0	0.03	0.03	0.001	0.34
C17:0	0.90[a]	0.82[b]	0.02	0.04

Supplemental Table S1. Continued.

**Parameter**	**Treatment**		**SEM**	***p*-value**
	**CNT**	**BIC**		
iso C18:0	0.07	0.06	0.004	0.06
C17:1 *cis* 6-7	0.02	0.02	0.02	0.46
C17:1 *cis* 8	0.06	0.06	0.002	0.20
C17:1 *cis* 9	0.29	0.25	0.01	0.07
C18:0 (SA)	9.55	10.1	0.25	0.10
C18:1 *trans* 4	0.01	0.01	0.001	0.10
C18:1 *trans* 5	0.01	0.01	0.001	0.03
C18:1 *trans* 6-8	0.19[b]	0.21[a]	0.007	0.04
C18:1 *trans* 9	0.17	0.18	0.006	0.26
C18:1 *trans* 10	0.28	0.30	0.02	0.48
C18:1 *trans* 11 (VA)	0.80	0.80	0.04	0.97
C18:1 *trans* 12	0.23	0.25	0.01	0.24
C18:1 *trans* 13 *trans* 14	0.63	0.67	0.04	0.49
C18:1 *cis* 9	19.6	18.7	0.69	0.27
C18:1 *cis* 11	0.38	0.36	0.01	0.23
C18:1 *cis* 12	0.16	0.17	0.008	0.54

C18:1 *cis* 13	0.05	0.05	0.002	0.31
C18:1 *trans* 16 *cis* 14	0.33	0.34	0.01	0.40
C19:0/C18:1 *cis* 15	0.25	0.24	0.01	0.56
C18:2 *trans* 10 *trans* 14	0.02[b]	0.03[a]	0.002	<0.001
C18:2 *trans* 11 *trans* 15	0.01	0.01	0.002	0.50
C18:2 *trans* 9 *trans* 12	0.01	0.01	0.001	0.60
C18:2c9 *trans* 13	0.30	0.28	0.01	0.39
C17cyclo	0.11	0.13	0.01	0.09
C18:2 *trans* 8 *cis* 13	0.14	0.13	0.005	0.67
C18:2c9 *trans* 12	0.09	0.09	0.002	0.50
C18:1 *cis* 16	0.02	0.02	0.001	0.99
C18:2 *trans* 9 *cis* 12	0.02	0.02	0.001	0.45
C18:2 *trans* 11 *cis* 15	0.15	0.13	0.01	0.36
C18:2 n-6 (LA)	2.18[a]	1.90[b]	0.07	0.02
C18:2 *trans* 12 *cis* 15	0.04	0.04	0.001	0.90
C18:2 *cis* 12 *cis* 15	0.01	0.01	0.001	0.58
C20:0	0.27	0.27	0.007	0.41

Supplemental Table S1. Continued.

**Parameter**	**Treatment**		**SEM**	***p*-value**
	**CNT**	**BIC**		
Δ7,9 17:2	0.02	0.02	0.001	0.38
C18:3 n-6	0.06[b]	0.08[a]	0.004	0.01
C20:1 *cis* 9	0.01	0.01	0.0003	0.32
C20:1 *cis* 11	0.04	0.04	0.002	0.36
C18:3 *n*-3 (LNA)	0.77[a]	0.59[b]	0.02	<0.001
CLA *cis* 9 *trans* 11 (RA)	0.45	0.43	0.02	0.33
C20:1 *cis* 15	0.01	0.01	0.001	0.65
CLA *trans* 9 *cis* 11/C21:0	0.09	0.09	0.004	0.95
CLA C18:2 *trans* 10 *cis* 12	0.007	0.008	0.0007	0.67
CLA *trans* 12 *trans* 14	0.01	0.01	0.001	0.10
CLA *trans* 11 *trans* 13	0.02	0.02	0.001	0.15
C20:2n9	0.02	0.02	0.001	0.51
CLA *trans* 9 *trans* 11	0.04[b]	0.05[a]	0.003	0.05
C18:4 n-3	0.01	0.01	0.001	0.90
C20:2 n-6	0.02	0.02	0.001	0.45

C20:3 n-9	0.05	0.05	0.003	0.52
C22:0	0.14	0.14	0.01	0.99
C20:3 n-6	0.03	0.03	0.001	0.80
10,14,17 C20:3	0.01	0.01	0.001	0.26
C22:1 n-9	0.01	0.01	0.001	0.52
C20:3 n-3	0.01	0.01	0.0005	0.71
C20:4 n-6	0.17	0.15	0.01	0.11
C23:0	0.07	0.07	0.04	0.79
C20:4 n-3	0.01	0.01	0.0002	0.32
C22:2 n-6	0.13	0.12	0.01	0.11
C20:5 n-3 (EPA)	0.07	0.06	0.003	0.07
C24:0	0.06	0.06	0.003	0.45
C22:3 n-6	0.01	0.01	0.0003	0.26
C24:1 *cis* 15	0.03	0.02	0.002	0.47
C22:4 n-6	0.01	0.01	0.001	0.38
C25:0	0.01	0.01	0.001	0.86
C26:0	0.04	0.04	0.003	0.58
C22:5 n-3 (DPA)	0.11^{a}	0.09^{b}	0.003	<0.01
C22:6 n-3 (DHA)	0.02	0.02	0.002	0.52

[a,b] Means within a row with different superscripts differ ($P \leq 0.05$).

[1] SA = stearic acid; VA = vaccenic acid; LA = linoleic acid; LNA = α-linolenic acid; RA = rumenic acid; EPA = eicosapentaenoic acid; DPA = docosapentaenoic acid; DHA = docosahexaenoic acid.
